# Protective Effects of *Mesembryanthemum Crystallinum* Extract Against Cadmium-Induced Reproductive Oxidative Stress: Experimental and Docking Evidence for a Sustainable Therapeutic Strategy

**DOI:** 10.1007/s12011-026-04975-0

**Published:** 2026-02-04

**Authors:** Hagar E. Mohammed, Ali El-Far, Shymaa Sobhy Mourad, Mai Alaa El-Dein, Menna H. E. Morsy, Mahmoud Bassiony, Shaimaa A. Hamouda

**Affiliations:** 1 Zoology Department, Faculty of Science, Arish University, Al-Arish, North Sinai 45511 Egypt; 2 Biochemistry Department, Faculty of Veterinary Medicine, Damnhour University, Damnhour, 22511 Egypt; 3 Zoology Department, Faculty of Science, Mansoura University, 35561 Mansoura, Egypt; 4 Faculty of Medicine, Alexandria University, Alexandria, Egypt

**Keywords:** Cadmium chloride, CAZA, Male fertility, *Mesembryanthemum crystallinum*, Oxidative stress, Sustainable development goals

## Abstract

One hazardous heavy metal that causes oxidative stress is cadmium (Cd) and harms male reproductive function. Although various antioxidants have been explored for protection, the potential of *Mesembryanthemum crystallinum* (*M. crystallinum*) – a flavonoid and phenolic-rich halophyte- remains understudied in this context. According to the United Nations Sustainable Development Goals (SDGs), specifically SDG 3 on Good Health and Well-Being and SDG 12 Responsible Consumption and Production this novel research evaluated the protective potential of *M. crystallinum* aqueous extract (MAE) to mitigate cadmium chloride (CdCl_2_)-induced testicular dysfunction. After acclimatization, Eight groups (*n* = 5) of forty male rats were allocated into groups: (1) control; (2) CdCl₂ (2 mg/kg); (3) MAE 200 mg/kg; (4) MAE 400 mg/kg; (5) CdCl₂ + MAE 200 mg/kg; (6) CdCl₂ + MAE 400 mg/kg; (7) MAE (200 mg/kg) pre-treatment + CdCl₂; (8) MAE (400 mg/kg) pre-treatment + CdCl₂. For 14 days, oral treatments were administered. HPLC profiling revealed eight major phenolic compounds in *M. crystallinum* like quercetin and ellagic acid. CdCl_2_ exposure impaired sperm indices, reduced serum LH and testosterone and induced oxidative and histological damage with elevated caspase-3 and TNF-*α* expression. MAE treatment especially at 400 mg/kg restored hormonal levels, improved sperm quality, normalized antioxidant defense, and preserved testicular architecture. Molecular docking confirmed binding of hesperidin, rutin, and ellagic acid to apoptotic and inflammatory proteins. MAE may exhibit potent anti-inflammatory, antioxidant, and anti-apoptotic activity, protecting against CdCl₂-induced reproductive injury. *M. crystallinum* represents a sustainable, plant based therapeutic candidate aligned with SDG-driven strategies for combating environmental toxicity-related infertility.

**Supplementary Information:**

The online version contains supplementary material available at 10.1007/s12011-026-04975-0.

## Introduction

 Heavy metals pollution, particularly from cadmium (Cd), poses a major global environmental and public health challenge due to its high level of toxicity and persistent bioaccumulation within living organisms. Cd is a non-essential heavy metal, widely recognized as a harmful environmental contaminant with no known biological functions [1].

The uptake of Cd by humans and animals is mostly through foods. All food ingredients contain Cd, even in small amounts. Some of the foods which have high levels of Cd are liver and offal, shellfish, mussels, seaweed, mushrooms, cocoa powder. Smoking also causes a high amount of chronic Cd exposure. Hazardous waste zones, factories that release Cd through air, and the metal refining industry are also important areas of Cd exposure [2, 3].

Chronic exposure to Cd—via industrial sources, contaminated food and water, or cigarette smoke—results in its accumulation in vital organs, including the liver, kidneys, and reproductive tissues, where its long biological half-life exacerbates its toxic effects [4]. Among its systemic effects, the impact of Cd on the fertility of male is particularly alarming [5, 6]. In experimental models, cadmium chloride (CdCl₂) is commonly employed to induce reproductive toxicity. In the reproductive male system, it impairs function of the testicles by inducing oxidative stress, weakening antioxidant defenses, and damaging testicular cells. This oxidative stress causes DNA damage, apoptosis and lipid peroxidation, which ultimately result in disrupted spermatogenesis, lowered sperm quality, and decreased fertility [7, 8]. These harmful effects emphasize the critical need for safe, effective protective strategies.

In recent years, growing interest in natural and sustainable therapeutics has aligned with the United Nations blueprint for Sustainable Development by2030. SDG3 (Good Health and Well Being) emphasizes affordable and accessible health care solutions, while United Nations SDG 12, which promotes Responsible Consumption and Production encourage the use of renewable biological resources in place of synthetic drugs [9, 10].

Traditional medicinal plants represent a crucial and overlooked pathway toward achieving these goals. Cultivated locally and sustainably, such plants not only support human heath but also strengthen environmental and economic resilience [11].

*Mesembryanthemum crystallinum* (*M. crystallinum*), typically known as the ice plant, is a climate resilient halophytic species abundant in North Siani, Egypt. It has gained recognition for its extensive content of flavonoids, phenolic acids and other phytochemicals with potent antioxidant and anti-inflammatory properties [12, 13]. Traditionally used in food, medicine, and cosmetics [14], its resilience to heavy metal stress has been documented, yet its specific protective role against CdCl₂- induced male reproductive toxicity, remains insufficiently explored, especially through mechanistic validation such as molecular docking. This research introduces a pioneering investigation into the protective role of *Mesembryanthemum crystallinum* extract against cadmium-induced oxidative stress specifically targeting reproductive toxicity, an underexplored area despite the plant’s established tolerance to heavy metals like cadmium. The novelty lies in its dual experimental and molecular docking evidence, which elucidates bioactive mechanisms for mitigating fertility impairment, extending beyond general antioxidant properties reported in prior studies on the plant. By framing this as a sustainable therapeutic strategy, the work aligns plant-based remediation with practical health applications, offering fresh insights for eco-friendly interventions in metal toxicity.

## Materials and Methods

### Ethical Considerations

Every experimental procedure was accepted by the committee on Animal Care and Ethics (ACEC) of Arish University, North Sinai, Egypt, (approval code: ARU004), and conducted with accordance with NIH protocols for the care and uses of animals in laboratories (NIH Publication No. 8023, updated 1978). Attempts were made to reduce animal pain and discomfort throughout the experiment, following ACEC recommendations.

### Reagents and Chemicals

Cadmium chloride (CdCl2; CAS No 10108-64-2) was bought from Sigma-Aldrich (St. Louis, MO, USA). ELISA kits for FSH, LH and testosterone were obtained from Biocheck, Inc, USA (catalog Numbers: MBS2021901, MBS764675 and MBS282195, respectively) in compliance with the manufacturer’s instructions. Kits for glutathione peroxidase (GPx), superoxide dismutase (SOD), catalase (CAT), and reduced glutathione (GSH) were purchased from Biodiagnostic (Cairo, Egypt; catalog numbers: 2524, 2521, 2511, and 2517, respectively). Malondialdehyde (MDA) kit was purchased from OxisResearch BIOXYTECH MDA-586 kit (Catalog No. 21044; USA). All other chemicals were of analytical grade.

### Plant Authentication and Processing

**Fig. 1:**
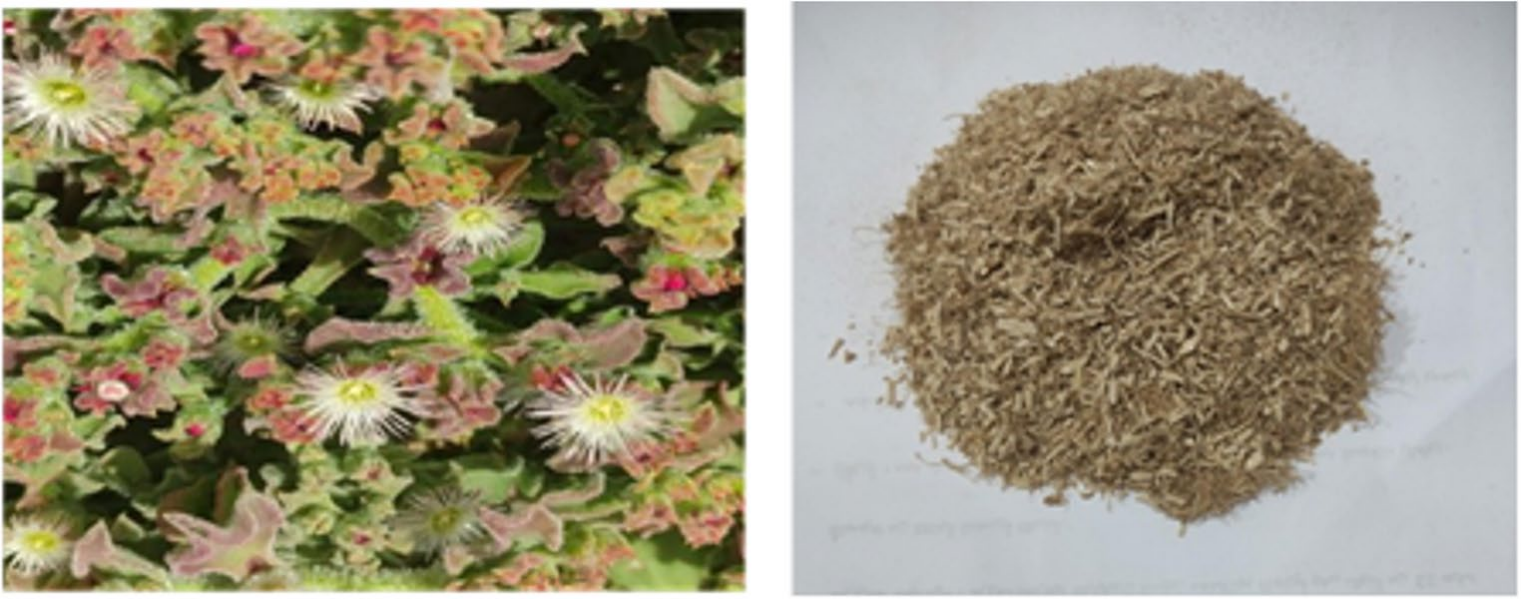
*Mesembryanthemum crystallinum* (**A**) plant and (**B**) powder

During May 2023, *M. crystallinum* plant was gathered at Al-Arish from the Northern Sinai desert, Egypt. The harvested edible portions of the plant were packaged and brought under chilled conditions to the labs. The identity of the plant was confirmed, and voucher samples were stored in the Faculty of Science’s Herbarium at Arish University, Egypt. Accession number A4483 was assigned to the specimens. The plants were oven dried at 55 °C for 24 h after being cleaned with distilled water. Subsequently, the material was grounded into a fine powder as shown in Fig. 1. and kept until analysis at a low temperature of 4 °C.

### Hot Water Extract Preparation

500 mL of distilled water with 250 g of fine powder, heated for 15 min, and then filtered. The filtrate was freeze-dried for further use [15].

### HPLC Examination

The flavonoid and phenolic constituents of *M. crystallinum* extract were analyzed via Agilent 1100 HPLC system (Santa Clara, CA, USA). All prepared samples, reference standard solutions, and mobile phase solvents were subjected to filtration via a 0.45 μm Millipore membrane filter and degassed before injection. A reversed-phase C18 column (2.5 × 30 cm) with an injection volume of 20 µL was used for chromatographic separation. The mobile phase was composed of solvent A (0.05% trifluoroacetic acid in acetonitrile) and solvent B (water), delivered using a gradient elution program as follows: 18.0% A and 82.0% at 0.000 min; 20.0% A and 80.0% at 5.0% A and 60.0% at 12.0 min, and 18.0% A and 82.0% at 20.0 min. The rate of flow was kept up at 1.0 ml/min, and the column temperature was set to 25 ◦C. Measurements was carried out using a diode array detector with a wavelength of 230–400 nm. Compound identification was achieved by matching their retention times and UV absorption spectra with those of authenticated standards [16].

### Experimental Design and Dosing Protocol

Male rats weighing between 160 and 250 g were kept in individually in cages with adequate ventilation and standard sanitary laboratory conditions. The animals were randomly assigned into eight experimental sets, each with 5 rats. Group 1 was used as normal control, which received normal saline. Group 2 was treated with CdCl₂ at a dose of 2 mg/kg body weight [17]. Groups 3 and 4 received *M. crystallinum* aqueous extract (MAE) at doses of 200 mg/kg and 400 mg/kg body weight, respectively. The higher dose was chosen based on a previous study by [18] that demonstrated its therapeutic potential. To investigate a potential dose dependent effect, a lower concentration was also chosen, representing half of the effective higher dose. Group 5 was co-administered CdCl₂ and MAE (200 mg/kg), while group 6 was co-administered CdCl₂ and MAE (400 mg/kg). To evaluate the prophylactic effect, groups 7 and 8 were pre-treated with MAE at 200 mg/kg and 400 mg/kg body weight, respectively, for one-week prior CdCl₂ administration, then MAE treatment continued daily until the conclusion of experimental period. Animals in the respective groups administered all designated treatments orally once every day via gavage. The dosage of CdCl₂‎ and MAE were determined according to the body weight of every rat. For a full description of the eight experimental groups and their treatments, see Supplement 1.

### Experimental Procedure and Sample Collection

The animal weights were documented at both the initiation and completion of the experiment. Percentage Weight Gain/Loss was calculated by the formula, (BW_final - BW_initial) / (BW_initial) × 100. Upon termination of the study, the animals were euthanized under isoflurane anesthesia and blood samples were obtained by cardiac puncture into plain tubes. Then allowed to clot and subsequently centrifuged to separate serum until required for the hormonal assay. The cauda epididymis was dissected immediately for sperm analysis. Both testes were removed and weighed. The absolute testis weight was divided by the final body weight (×100) to calculate the relative testis weight. The left testis was homogenized in phosphate buffer (pH 7.4), and resulting clear liquid was used to assess oxidative stress biomarkers. MDA levels were assessed using the method described by Ohkawa [19], while reduced GSH was determined using the colorimetric technique illustrated by Ellman [20], The concentration of GPx, SOD and CAT were quantified spectrophotometrically following the protocol of Weydert and Cullen [21]. The right testicle was preserved in Bouin’s solution for subsequent histopathological and immunohistochemical examinations.

The hormonal assays luteinizing hormone (LH), testosterone and follicle-stimulating hormone (FSH) were quantified using ELISA commercial kits, following the protocol of manufacturer’s instructions for calculation and interpretation.

### Sperm Kinematics Using CASA System (CFT-9200)

Semen parameters were assessed using the Sperm Vision™ computer-assisted analysis System (CASA; MiniTüb, Germany) with an Olympus BX 51 phase contrast microscope (Olympus, Japan). Immediately after dissection, the cauda epididymis was carefully dissected out, minced with surgical scissors in 2 ml of 0.9% physiological saline, and then incubated at 37 °C for 20 min to allow spermatozoa to leave the epididymal tubules. A 10 mL aliquot was pipetted and placed on a slide to evaluate the sperm parameters. The velocity average path (VAP; µm/s), velocity straight line (VSL; µm/s), velocity curved line (VCL; µm/s), straightness (STR; %), wobble (WOB; %), linearity (LIN; %), mean angular degree (MAD; ^°^), beat cross frequency (BCF; Hz) and amplitude of lateral head displacement (ALH; µm) were analyzed according to the protocol of manufacturer’s protocol.

### Morphology and Sperm Normality Criterion

A small aliquot of sperm sample was spread onto a glass slide and fixed with methanol. After air drying for 10 minutes’ slides were colored with 2% Giemsa. Stained preparations were examined under light microscope (Olympus IX-71, Tokyo, Japan) at a magnification of ×400. Morphological assessment was performed on a monitor screen. Normal sperm morphology was characterized by an oval head, intact neck, sidepiece, and tail with hook-shaped heads and thin uniform tail. Abnormal spermatozoa included headless and hookless cells; amorphous forms; folded or shortened, and double Y tail, and other irregularities.

### Histopathological and Immunohistochemical Analysis

Following fixation in Bouin’s solution, the testes were immersed in 70% ethanol for preservation. Subsequently, the samples underwent dehydration through a graded ethanol series, processed in xylene, and vertically inserted in paraffin wax for sectioning. 

Specimens that were 4 μm thick were sliced using rotor microtomes. After paraffin slices were processed in xylene for 20 min to eliminate paraffin and dried in a succession of increasingly diluted ethanol solutions, they underwent hematoxylin and eosin (H&E) staining [22]. The extent of testicular histological injury measured using Johnsen’s testicular score method. Thirty cross-sectioned tubules were evaluated for each group, and every tubule was assigned a level ranging from 1 (very bad- No seminiferous epithelium) to 10 (excellent-Full spermatogenesis) based on Johnsen’s criterion [23].

For immunostaining, wax was removed and hydrated testicular slices were rinsed for 15 min in PBS, followed by a 10-minute processing with 0.3% hydrogen peroxide to block the activity of endogenous peroxidase. Immunostaining was performed using the streptavidin-biotin immunoperoxidase protocol. Rabbit polyclonal antibodies against caspase-3 (ab13847; Abcam) and mouse monoclonal antibodies against tumor necrosis factor-alpha (TNF-*α*; Code: CSB-E04740h, CUSABIO) were utilized. Primary antibodies were incubated on sections overnight at 4 °C; thereafter, Image J software was used to evaluate the labeled index [24]. ImageJ (NIH) software was used for quantification and standardized thresholding followed by measurement of area % and mean intensity.

.

### Molecular Docking Assessment

The three-dimensional structures of rats’ follicle-stimulating hormone receptor (FSHR), luteinizing hormone receptor (LHR), androgen receptor, TNF-*α*, and caspase-3 were retrieved from the UniProt (https://www.uniprot.org/) database. The three-dimensional composition of cadmium chloride was sourced from the PubChem archive (https://pubchem.ncbi.nlm.nih.gov/). Computational docking assess-ment between proteins and ligands was done using UCSF Chimera and Autodock Vina software. Besides, BIOVIA Discovery Studio Visualizer 2016 software visualized their molecular interactions.

### Statistical Analysis

Findings were processed in GraphPad Prism 8.0.1 software (San Diego, CA, USA) and are expressed as mean ± SEM (*n* = 5). One-way ANOVA with Tukey’s post hoc test was conducted to compare differences between sets. Statistical significance was set as ^*^P < 0.05 as opposed to the control. On the other hand, ^**#**^*P* < 0.05 when compared to the CdCl₂ group.

## Results

### Retention Time and Peak Analysis of Phenolics by HPLC

**Fig. 2:**
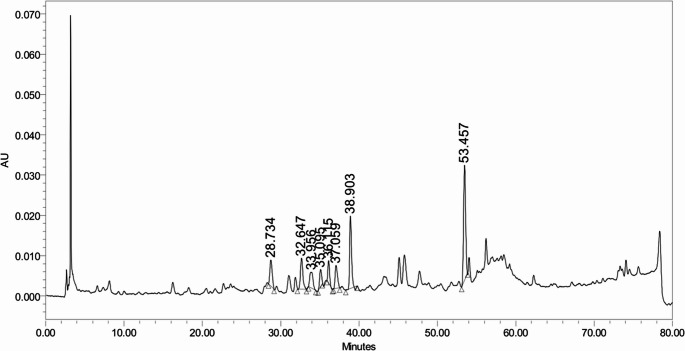
HPLC chromatogram of phenolic compounds identified in methanolic extract of *M. crystallinum*

HPLC chromatogram analysis revealed the presence of eight major phenolic compounds in the plant sample, based on their retention times (Fig. 2). The chromatographic data are presented in Table 1, detailing the peak name, retention time (RT), peak area, percentage of the total area, and peak height. Quercetin (34.46%) and ellagic acid (20.52%) are the most dominant phenolic compounds and collectively constitute over half of the total phenolic content measured. This indicates that these two compounds are the primary bioactive constituents. Other compounds, such as catechin (10.67%), gallic acid (8.52%), and rutin (7.9%), are found in moderate quantities. Chlorogenic acid (7.33%), hesperidin (5.62%), and caffeic acid (4.98%) are present in lower concentrations compared to quercetin and ellagic acid.

**Table 1:** Chemical composition analysis of phenolic compounds in methanolic extract of *M. crystallinum* by HPLC

No.	Peak Name	RT	Area	% Area	Height
1	Gallic acid	28.734	129,981	8.52	6438
2	Catechin	32.647	162,678	10.67	7662
3	Chlorogenic acid	33.956	111,750	7.33	3949
4	Caffeic acid	35.095	75,940	4.98	4227
5	Hespiridin	36.115	85,756	5.62	5538
6	Rutin	37.059	120,518	7.90	5738
7	Ellagic acid	38.903	312,992	20.52	17,948
8	Quercetin	53.457	525,528	34.46	28,670

### Body and Testis Weight

Table 2 shows the body gain (BWG) and relative testes weight (RTW) of control and treated animals after 14 days. No statistically significant differences were detected in BWG or RTW among the control, MAE200-treated, and MAE400 groups. However, CdCl₂ exposure led to a marked drop in both BWG (*P* < 0.05) and RTW (*P* < 0.00) compared to control values. Co-intake of MAE at either 200 mg or 400 mg along with Cd markedly improved both parameters.

**Table 2:** Body weight Gain/Loss (%) and relative testicular weight (%) of male rats in control and treatment groups. Data indicates the mean ± S.E for observations from 5 rats. *p < 0.05 is significantly different from control group and ^#^*p* < 0.05 from CdCl_2_ group

Groups	BWG/L (%)	Relative Testicular weight (%)	
		**Right Left**	
NC	+ 14.56 ± 0.69	0.78 ± 0.01	0.76 ± 0.04
CdCl_2_	+ 7.7 ± 0.33^*^	0.53 ± 0.01^***^	0.49 ± 0.003^***^
MAE200	+ 15.38 ± 1.03	0.66 ± 0.37	0.67 ± 0.02
MAE400	+ 16.7 ± 0.6	0.71 ± 0.02	0.72 ± 0.02
CdCL_2_ + MAE200	+ 26.7 ± 1.33^###***^	0.64 ± 0.06^**###**^	0.69 ± 0.04^**###**^
CdCl_2_ + MAE400	+ 23.95 ± 0.95^###**^	0.59 ± 0.08^*##^	0.68 ± 0.008^**###**^
MAE200 + CdCl_2_	+ 23.1 ± 1.46^###**^	0.61 ± 0.01^**#^	0.7 ± 0.01^**###**^
MAE400 + CdCl_2_	+ 32.73 ± 1.26^###***^	0.6 ± 0.02^*#^	0.69 ± 0.03^**###**^

### Sex Hormones in Rats of Different Treated Groups

Table 3 illustrates the serum hormonal profile following cadmium exposure and treatment. All hormonal assays were performed in a duplicate and validating using internal controls and manufactured calibration curves. CdCl₂ administration resulted in marked endocrine disruption, evidenced by a significant drop in serum testosterone (*P* < 0.05) and LH level (*P* < 0.001) relative to the control rats. However, FSH level remained unchanged statistically across all treated groups. Simultaneous administration with MAE at either 200 mg or 400 mg significantly restored testosterone level toward control values, with the combined treatment showing superior potential in normalizing hormonal balance. Furthermore, the combined intake of both extracts to CdCl₂ group was more effective in normalizing the level of this hormone. These hormonal improvements paralleled the dose-dependent preservation of testicular architecture observed histologically.

**Table 3:** Determination of sex hormones in rats of different treated groups

Groups	FSH (mIU/ml)	LH (mIU/ml)	Testosterone (ng/ml)
NC	0.27 ± 0.01	0.34 ± 0.02	5.88 ± 0.07
CdCl_2_	0.25 ± 0.003	0.21 ± 0.001^***^	1.13 ± 0.03^*^
MAE200	0.26 ± 0.01	0.36 ± 0.005	9.58 ± 0.67
MAE400	0.25 ± 0.003	0.41 ± 0.008^*^	14.25 ± 1.53^***^
CdCl_2_ + MAE200	0.28 ± 0.01	0.37 ± 0.02^**####**^	13.56 ± 1.17^****####^
CdCl_2_ + MAE400	0.3 ± 0.01^**#**^	0.37 ± 0.02^**####**^	16.26 ± 0. 92^****####^
MAE200 + CdCl_2_	0.19 ± 0.01^***#^	0.42 ± 0.008^*####^	15.9 ± 1.42^****####^
MAE400 + CdCl_2_	0.22 ± 0.003^*^	0.39 ± 0.01^**####**^	16.23 ± 0.62^****####^

Data indicates the mean ± S.E for observations from 5 rats. *p < 0.05 is significantly different from control group and ^#^*p* < 0.05 from CdCl_2_ group

### Effect of MAE on Rat Sperm Motility

Table 4 summarizes the effects of CdCl₂, MAE, and their combinations on sperm kinetics in rats. Exposure to CdCl₂ significantly impaired sperm quality, indicated by marked reductions in sperm number, motility (*p* < 0.05), vitality (*p* < 0.01), and velocity parameters- VCL, MAD, LIN, and BCF (*p* < 0.05) against the control set. Treatment with MAE alone (200 and 400 mg/kg) exerts moderate effect on mobility and vitality in comparison to control group. MAE-treatment either concurrently or prior CdCl₂ exposure showed significant improvements in these parameters relative to CdCl₂ treated group. Notably, MAE200 + CdCl₂ group displayed the highest sperm count (8.99 ± 1.8), motility (44.35 ± 0.7%), and vitality (45.83 ± 0.8%), suggesting a potential prophylactic role. Furthermore, velocity-related parameters (VCL, MAD, LIN, and BCF) were better preserved in both pre-and concurrent-treated groups than in rats exposed to CdCl₂-alone.

**Table 4:** Sperm kinetics parameter for the control rat and the treated groups

Parameters	NC	CdCl_2_	MAE200	MAE400	CdCl_2_ + MAE200	CdCl_2_ + MAE400	MAE200 + CdCl_2_	MAE400 + CdCl_2_
Sperm Count (million/ml)	6.09 ± 1.5	1.94 ± 0.6^*^	5.93 ± 0.12	8.013 ± 0.5	3 ± 1.013	2.31 ± 0.87	8.99 ± 1.8^**###**^	6.91 ± 8.07^**##**^
Motility (%)	32.99 ± 4.86	21.13 ± 0.7^*^	43.95 ± 2.7*	38.33 ± 3.2^**^	47.78 ± 2.8^*#^	25.026 ± 1.6^**#^	44.35 ± 0.7*^#^	33.25 ± 1.44^*#^
Vitality (%)	25.36 ± 1.75	19.23 ± 0.94^**^	30.63 ± 2.44^**^	28.47 ± 1.01	39.16 ± 1.44^**#^	42.6 ± 1.98^*#^	45.83 ± 0.8*^#^	25.71 ± 2.5^**#^
VCL (µm/s)	8.56 ± 0.57	5.43 ± 1.05^*^	5.86 ± 0.87	6.88 ± 1.53	2.69 ± 0.77^****^	3.98 ± 1.37^**^	8.33 ± 1.04	8.48 ± 1.25^#^
VSL (µm/s)	1.45 ± 0.85	1.007 ± 0.41	0.91 ± 0.28	1.38 ± 0.16	1.45 ± 1.25	1.51 ± 0.82	1.55 ± 0.52	1.28 ± 0.64
VAP (µm/s)	3.52 ± 1.79	2.63 ± 0.7	1.62 ± 0.82	3.28 ± 0.75	1.33 ± 0.032	3.13 ± 2.12	3.67 ± 0.88	3.26 ± 0.91
MAD (^°^)	15.66 ± 9.24	9.09 ± 2.15^*^	15.75 ± 4.26	17.07 ± 4.24	15.83 ± 0.17	15.64 ± 0.53	25.51 ± 0.66^**####**^	27.34 ± 0.84^**####**^
ALH (µm)	3.89 ± 1.41	1.61 ± 0.34	1.78 ± 0.94	2.76 ± 0.5	2.11 ± 0.44	1.95 ± 0.44	3.58 ± 0.94	3.12 ± 1.76
BCF (Hz)	2.42 ± 1.17	1.41 ± 0.35	1.66 ± 0.84	1.79 ± 0.26	1.38 ± 0.75	1.62 ± 0.70	2.63 ± 0.48	2.42 ± 0.99
LIN %	7.45 ± 0.79	2.87 ± 0.2^*^	7.48 ± 0.62	8.2 ± 0.68	14.07 ± 0.43^**####^	13.49 ± 1.74^**####^	17.05 ± 1.123^****####^	16.64 ± 0.57^****####^
WOB%	17.74 ± 0.87	11.42 ± 0.59^*^	16.92 ± 1.16	17.32 ± 0.56	36.33 ± 2.38^****####^	28.64 ± 0.93^**####^	45.07 ± 3.57^****####^	39.91 ± 1.85^****####^
STR%	19.02 ± 0.83	15.07 ± 0.55	18.44 ± 0.06	17.6 ± 1.55	38.16 ± 5.8^**###^	23.23 ± 0.98	37.02 ± 1.29^**###^	41.75 ± 3.26^***####^

Data indicates the mean ± S.E for observations from 5 rats. *p < 0.05 is significantly different from control group and ^#^*p* < 0.05 from CdCl_2_ group

*DAP* (distance average path, mm), *DCL* (distance curved line, mm) *DSL* (distance straight line, mm), *VAP* (velocity average path, mm/s), *VCL* (velocity curved line, mm/s), *VSL* (velocity straight line, mm/s), *STR* (straightness, VSL/VAP, %), *LIN* (linearity, VSL/VCL, %), *WOB* (wobble, VAP/VCL, %), *ALH* (amplitude of lateral head displacement, mm), and *BCF *(beat cross frequency)

### Assessment of Sperm Morphological

**Fig. 3:**
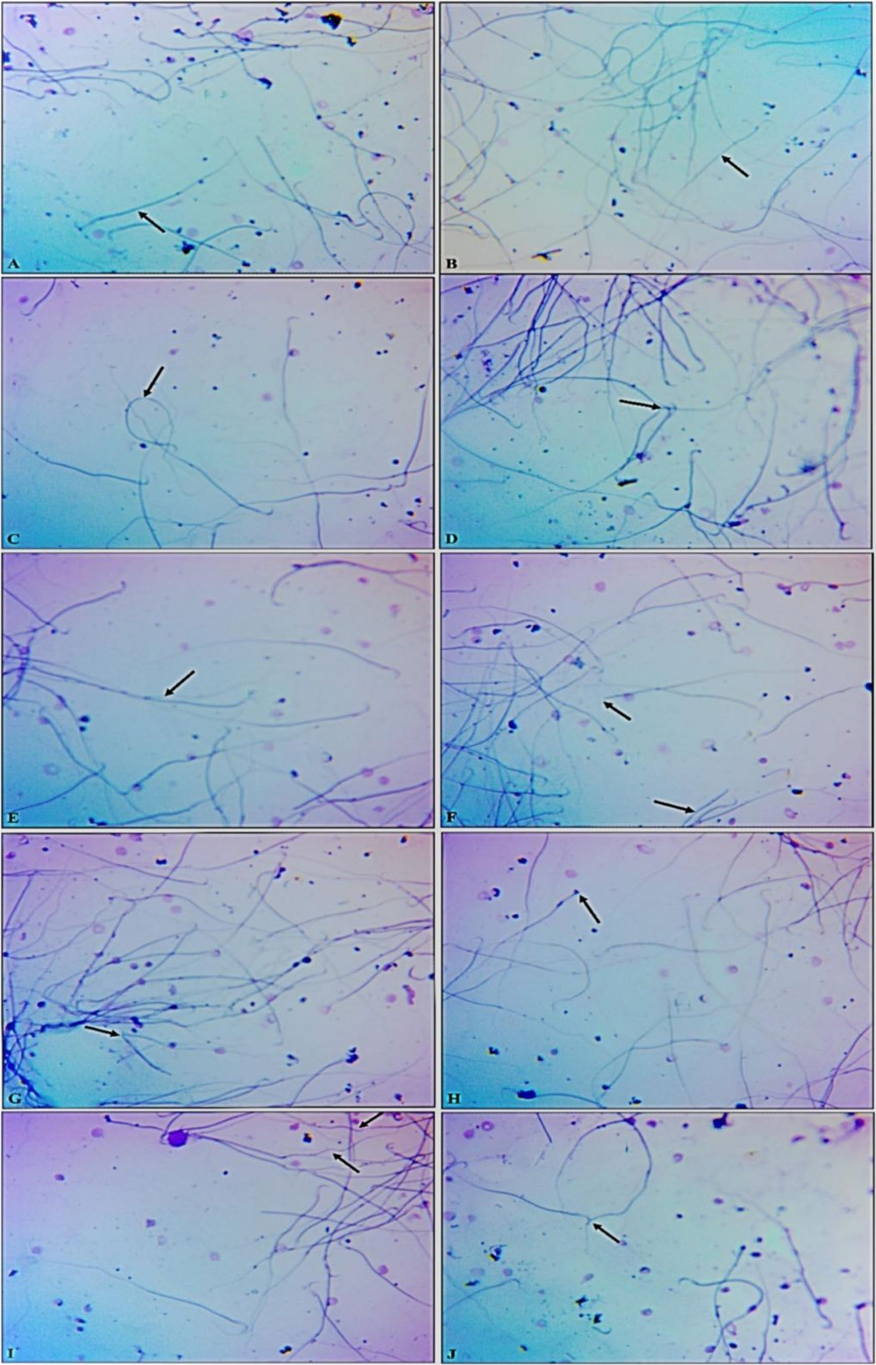
Microphotographs demonstrate morphologically normal sperm and numerous sperm defects. (**A** and **B**) Normal morphology. (**C**) Coiled tail. (**D**) Bent neck. (**E**) Double tail. (**F**) Headless and hairpin tail. (**G**) Headless and broken tail. (**H**) Headless tail. (**I**) Curved tail, (**J**) Pairing phenomenon

Sperm morphological abnormalities like bent and coiled tail, sperms without head, sperms with broken head, curved and hairpin tail of the experimental rats are presented in Fig. 3.

### Histopathological Investigation

**Fig. 4:**
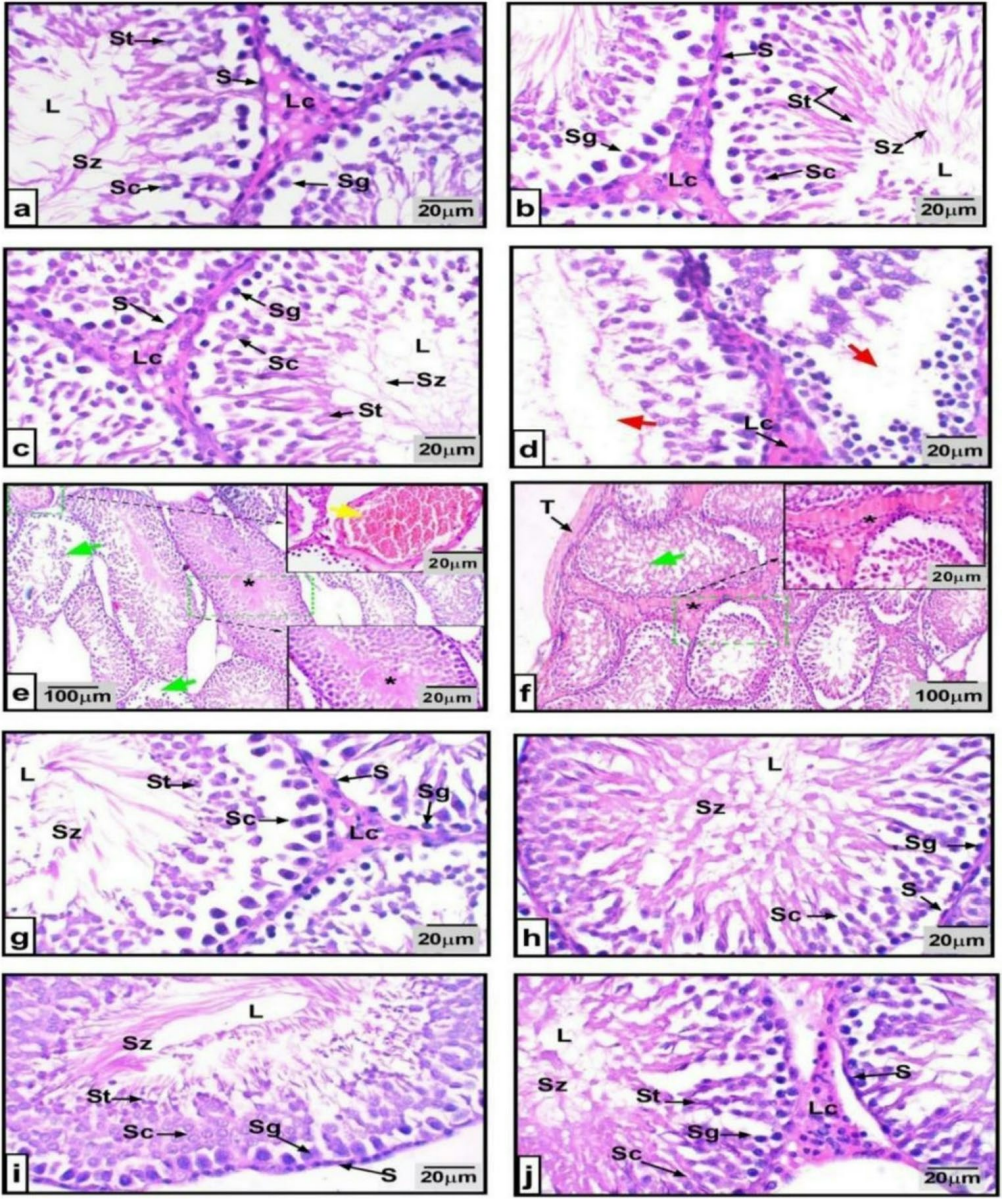
Effect of cadmium chloride (CdCl_2)_, MAE low-dose (200 mg/kg), and high-dose (400 mg/kg) on testicular histopathology: Control group **(a)**; MAE 200 group**(b)**; MAE high-dose group **(c)**, depicting typical Leydig cells (Lc), Sertoli cells (S), spermatogonia (Sg), spermatocytes, spermatids, and spermatozoa (Sz) in the seminiferous tubular lumen; **(d-f)**. Cdcl_2_ treated group; shows disorganized and degenerated germinal layers (red arrow), diffused Leydig cells (Lc); shows congested blood vessel (yellow arrow), depleted germ layer (green arrow), and occluded tubular lumen (asterisk); shows testicular fibrosis in tunica albuginea (T) and disorganized tubules (green arrow); **(g-h)** CdCl_2_ + MAE200 group; CdCl_2_ + MAE400 display almost normal tubules with complete spermatogenesis; **(i-j)** protected CdCl_2_ + MAE 200 group protected CdCl_2_ + MAE 400 group depicting that germinal cells were complete up to spermatids and full sperm maturation occurred. **H&E stain**. X100 for scale bar 100 μm. × 400. Scale bar = 20 μm

Testicular sections from all groups were examined for histopathological alteration. Normal, MAE 200 mg/k, and MAE400 mg/kg groups showed typical histoarchitecture of seminiferous tubules, with well – organized Leydig and Sertoli cells, and complete spermatogenesis stages **(**Fig. 4a-c**)**. In contrast, CdCl₂ administration revealed evident testicular malfunction, including germinal epithelium atrophy, disrupted spermatogenesis, vascular congestion, occluded tubular lumens, and fibrosis, extended into the tunica albuginea **(**Fig. 4d-f**)**. Co-treatment with MAE at both doses showed a remarkable improvement in the testicular architecture, especially in CdCl_2_ + MAE high-dose group **(**Fig. 4g and **h)**. Pretreatment with MAE at both doses also provided protective effects, maintaining near- normal germinal distribution and spermatogenesis processes **(**Fig. 4i and **j).**

**Fig. 5:**
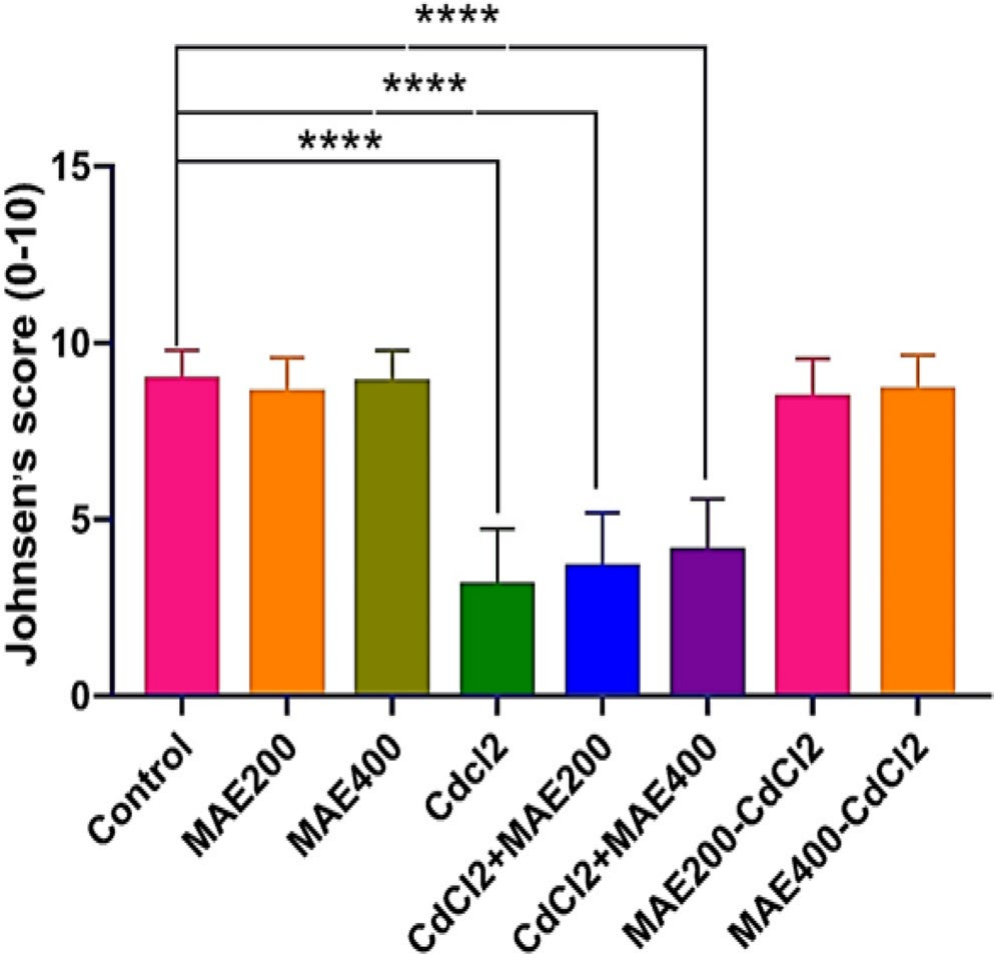
Depicts the normalized distribution of Johnsen scores across transverse sections of seminiferous tubules, comparing histological profiles between control and other treated groups. Johnsen’s score was significantly reduced in the CdCl_2_-affected groups, representing disturbed spermatogenesis

Spermatogenic integrity was quantitatively assessed using the Johnsen scoring system, which orders scores seminiferous tubules from 1 to 10 based on the degree of germ cell depletion and disruption of the seminiferous epithelium. Figure 5 presents the standard distribution of Johnsen scores across all experimental groups.

### Immunohistochemical Assessments (IHC)

**Fig. 6:**
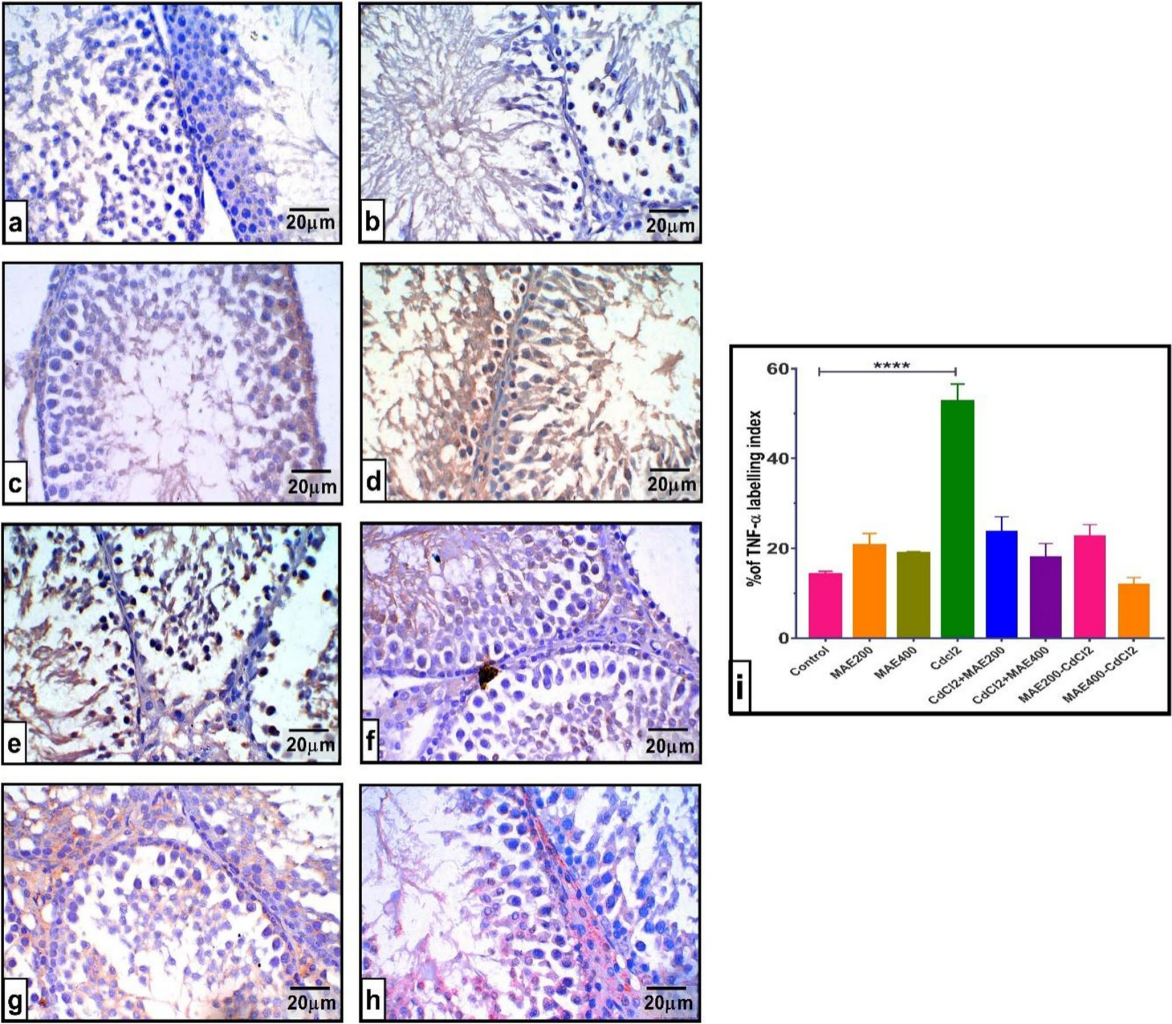
Effect of cadmium chloride (CdCl_2_) intoxication and MAE200, and MAE400 on testicular TNF-*α* ‎‎‎(immunohistochemically) of different groups: (**a**). Normal Control group; (**b**). MAE200 group; ‎‎(**c**). MAE400 group; ‎Revealing a mild TNF-*α* immuno-reactivity; (**d**). CdCl_2_ intoxication ‎group depicts intensive ‎expression of TNF-*α*‎; (**e**). CdCl_2_ + MAE200 group shows a moderate ‎expression of TNF-*α*; ‎‎(**f**). CdCl_2 _+ MAE400 ‎ group displays TNF-*α* ‎weak expression; (**g**). ‎MAE200 + CdCl_2_ group TNF-*α* exhibited mild immunoreactivity; A markedly reduced expression of TNF-*α* was observed in MAE400 + CdCl_2_ (**h**), indicating attenuated inflammatory signaling. (**i**) TNF-*α* ‎expression area%. Values are ‎expressed as means ± SEM; (*n* = 5 ‎microscopic fields/tissue samples of TNF-*α* immunostaining, 400X). ****p < 0.0001 vs. Normal control ‎group

IHC data were derived from blinded quantification and presented as mean area % and mean intensity. Testicular tissues from control animals and those administered with MAE at 200 and 400 mg/kg demonstrated a moderate TNF-α expression. CdCl_2_ intoxication significantly elevated TNF-*α* levels (*P* < 0.0001), with pronounced immunoreactivity observed in the tissue. Concurrent oral treatment of MAE at 200 and 400 mg/kg with CdCl_2_ decreased TNF-*α* expression, rendering it statistically insignificant in comparison to normal rats (Fig. 6e and **f**). Similarly, the exposure of MAE at 200 and 400 mg/kg to rats before CdCl_2_ exposure resulted in a mild, insignificant expression in testicular TNF-*α* levels relative to the normal group.

Parallel evaluation of caspase-3 expression demonstrated a comparable pattern. Control and MAE treated groups exhibited moderate caspase-3 immunoreactivity. CdCl_2_ intoxication markedly upregulated caspase-3 expression relative to control group (*p* < 0.001). However, co-oral exposure of MAE at 200 mg and 400 mg dramatically reduced caspase-3 levels (*p* < 0.001 and *p* < 0.05, respectively) relative to normal rats and normalized its tissue expression (Fig. 6e and **f**). Meanwhile, pre-administering MAE to rats at both doses before CdCl_2_ poisoning led to a statistically significant reduction in testicular caspase-3 contents (*p* < 0.001 and *p* < 0.01, respectively) against CdCl_2_ treated rats, indicating a protective effect.

### Oxidative Stress Biomarkers in Rats’ Testes

**Fig. 7:**
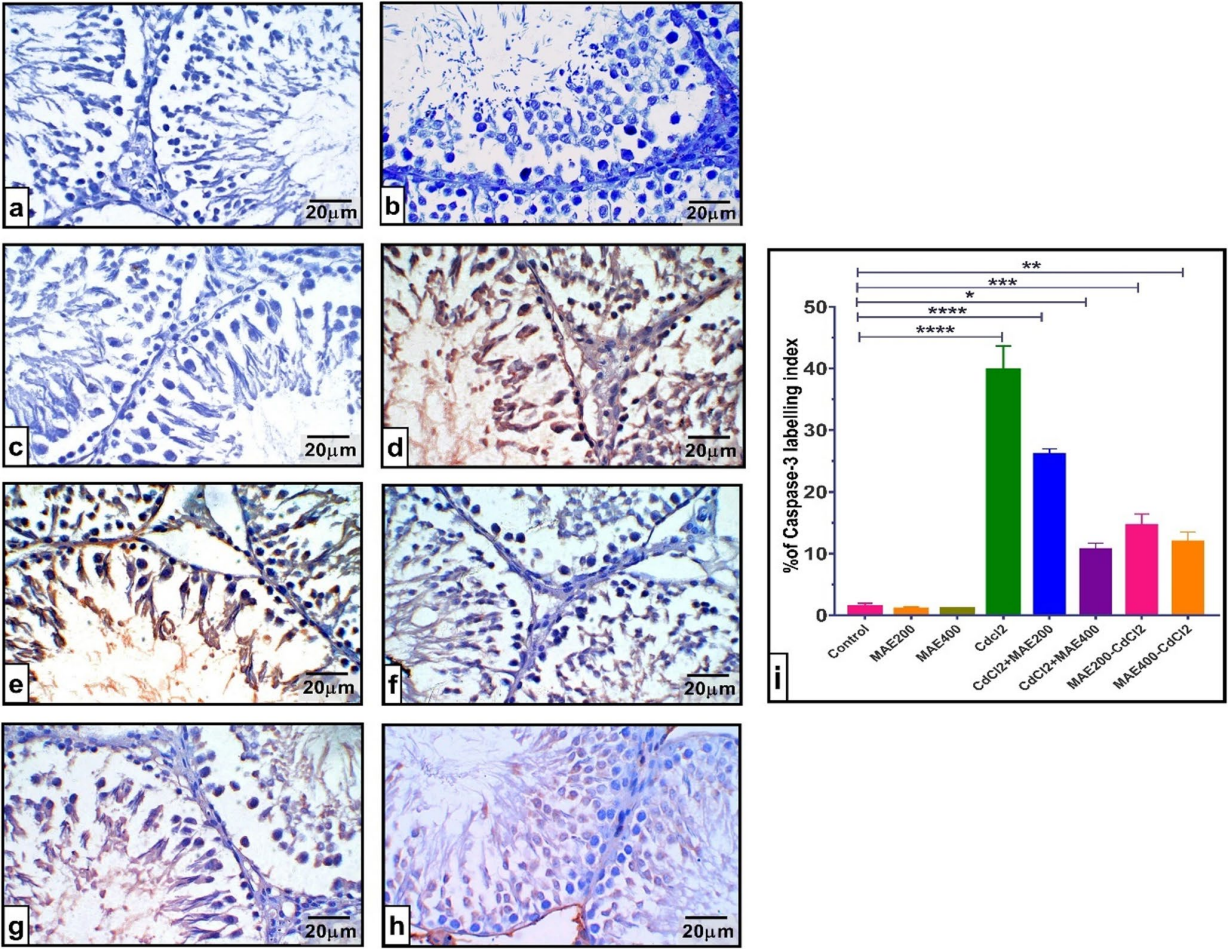
Effect of cadmium chloride (CdCl_2_) intoxication and MAE200, and MAE400 on testicular caspase-3 (immunohistochemically) of different groups: (**a**). Normal Control group; **(b**). MAE200 group; (**c**). MAE400 group; ‎Revealing a mild caspase-3 immuno-reactivity; (**d**). CdCl_2_ intoxication group depicts intensive ‎expression of caspase-3; (**e**). CdCl_2_ + MAE200 group shows a moderate expression of caspase-3; ‎‎(**f**). CdCl_2_ + MAE400 ‎group shows that caspase 3 expression was minimally detected via immunohistochemical analysis, revealing mild apoptotic activity. (**g**). MAE200 + CdCl_2_ demonstrates mild-level caspase-3 immunoreactivity; (**h**) MAE400 + CdCl_2_ exhibits a modest caspase-3 expression. (**i**) Caspase-3 expression area%**‎.** Values are expressed as means ± SEM; (*n* = 5 ‎microscopic fields/tissue samples of caspase-3 immune-expression, 400X). *p < 0.05, ‎**p < 0.01, ‎***p < 0.001, ****p < 0.0001 vs. Normal control group

**Fig. 8:**
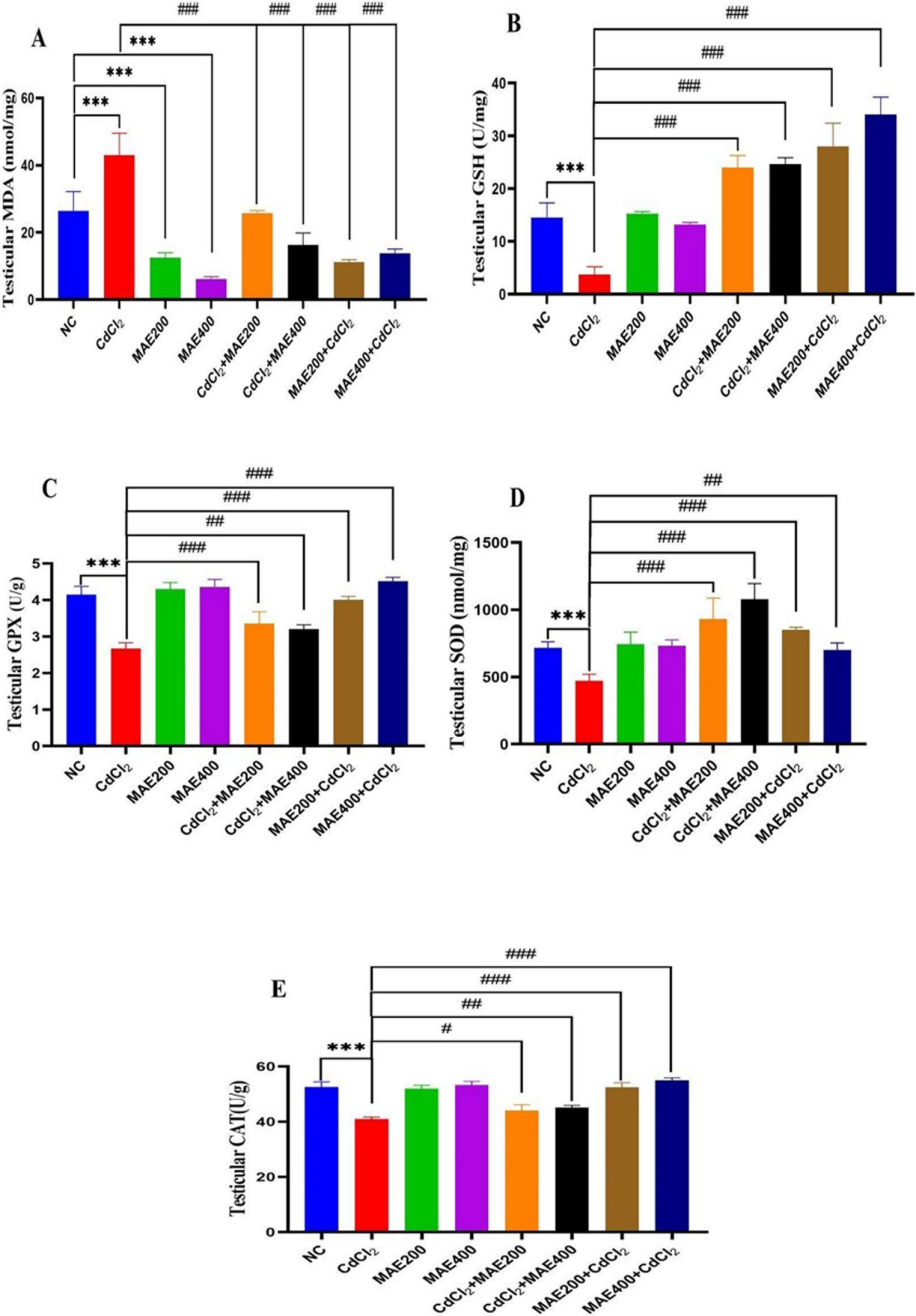
Malondialdehyde (MDA) and antioxidants profile between the control and different experimental groups. Data indicates the mean ± S.E for observations from 5 rats. *p < 0.05 is significantly different from control group and ^#^*p* < 0.05 from CdCl_2_ group

Figure 8 illustrates the levels of MDA and key antioxidant markers in testicular tissues across experimental groups. CdCl₂ exposure significantly increased MDA levels and depleted the contents of GSH, GPx, CAT and SOD (*p* < 0.001 vs. control), indicating pronounced oxidative stress. Both co- and pre-treatment with MAE at 200 and 400 mg/kg significantly lowered MDA concentrations, with pre-treatment providing the most pronounced protective effect (Fig. 7A to 8A**)**. Additionally, MAE administration- whether prior to or alongside CdCl₂ exposure, at both doses, effectively restored antioxidant levels (*p* < 0.001 vs. CdCl₂ group), reflecting a strong antioxidant defense against CdCl₂-induced testicular oxidative stress (Fig. 8).

### Molecular Docking Interactions

**Fig. 9:**
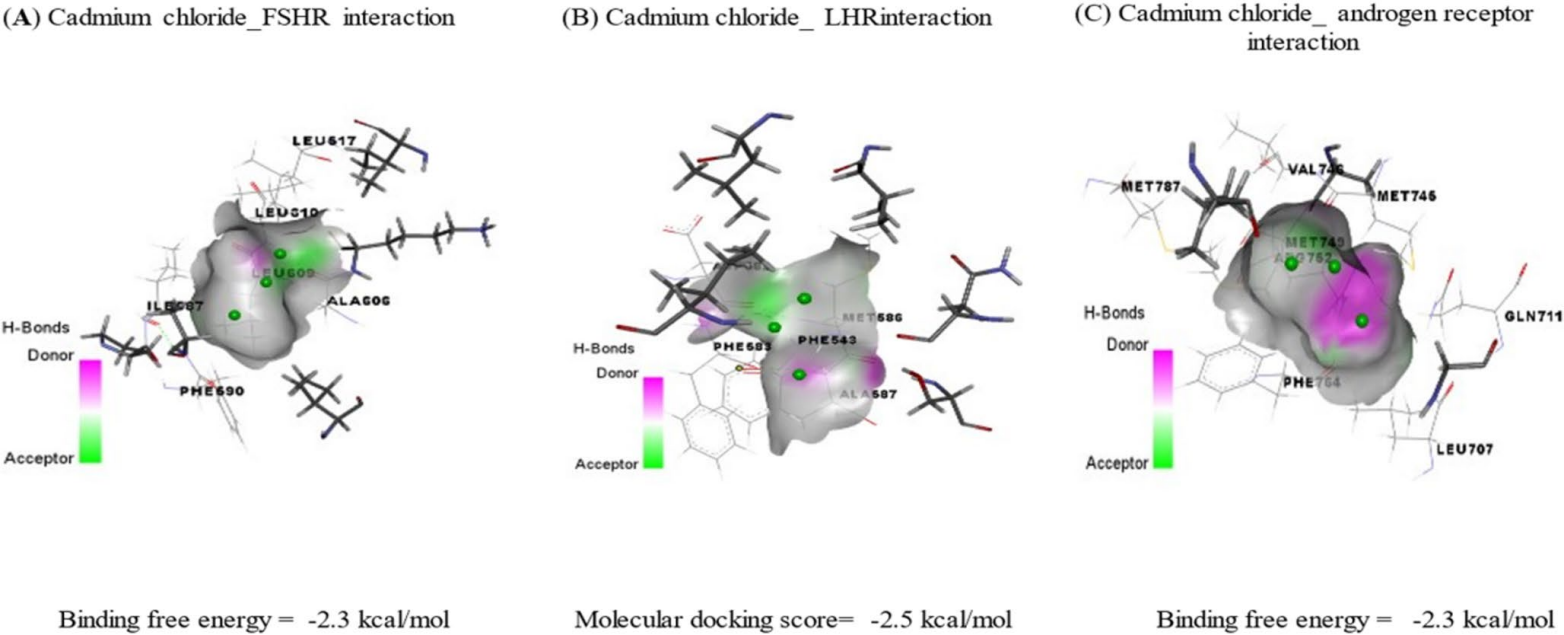
Molecular docking of cadmium chloride with rats’ follicle-stimulating hormone receptor (FSHR), luteinizing hormone receptor (LHR), and androgen receptor

**Fig. 10:**
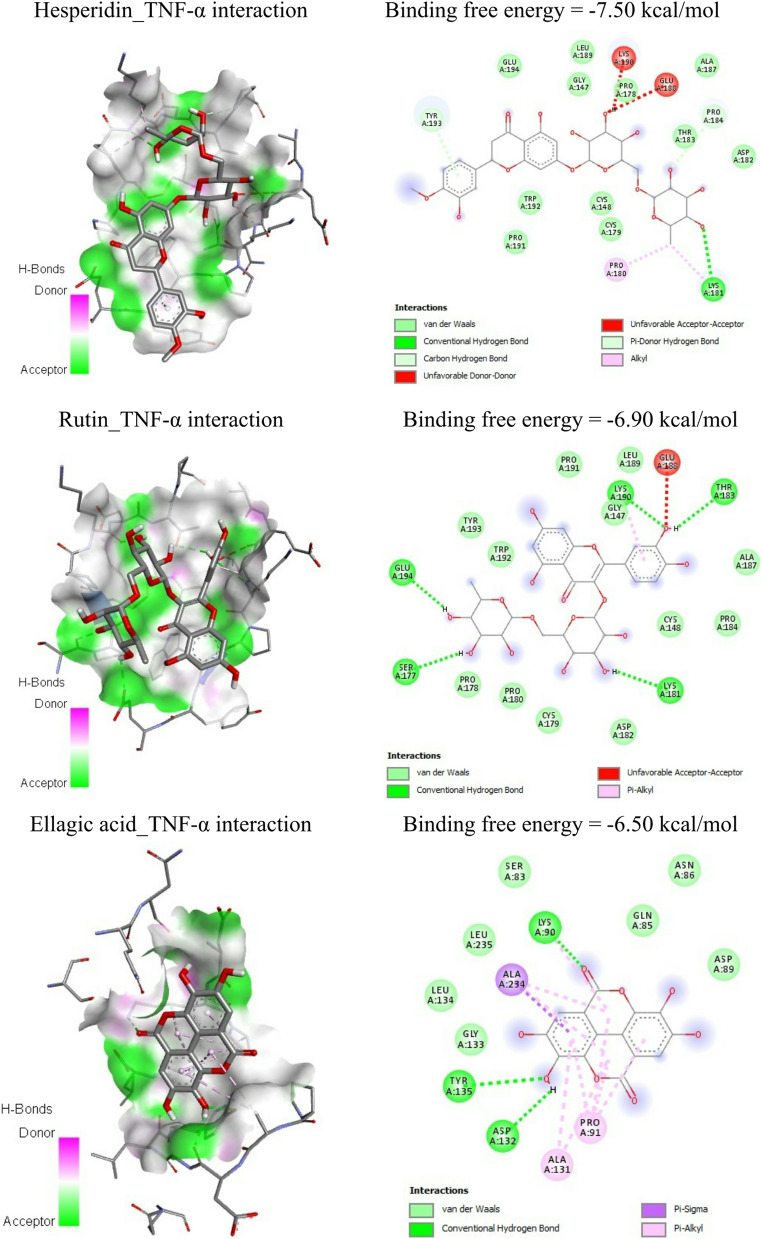
Molecular docking binding free energies of (**A**) hesperidin, (**B**) rutin, and (**C**) ellagic acid against TNF-*α*

**Fig. 11:**
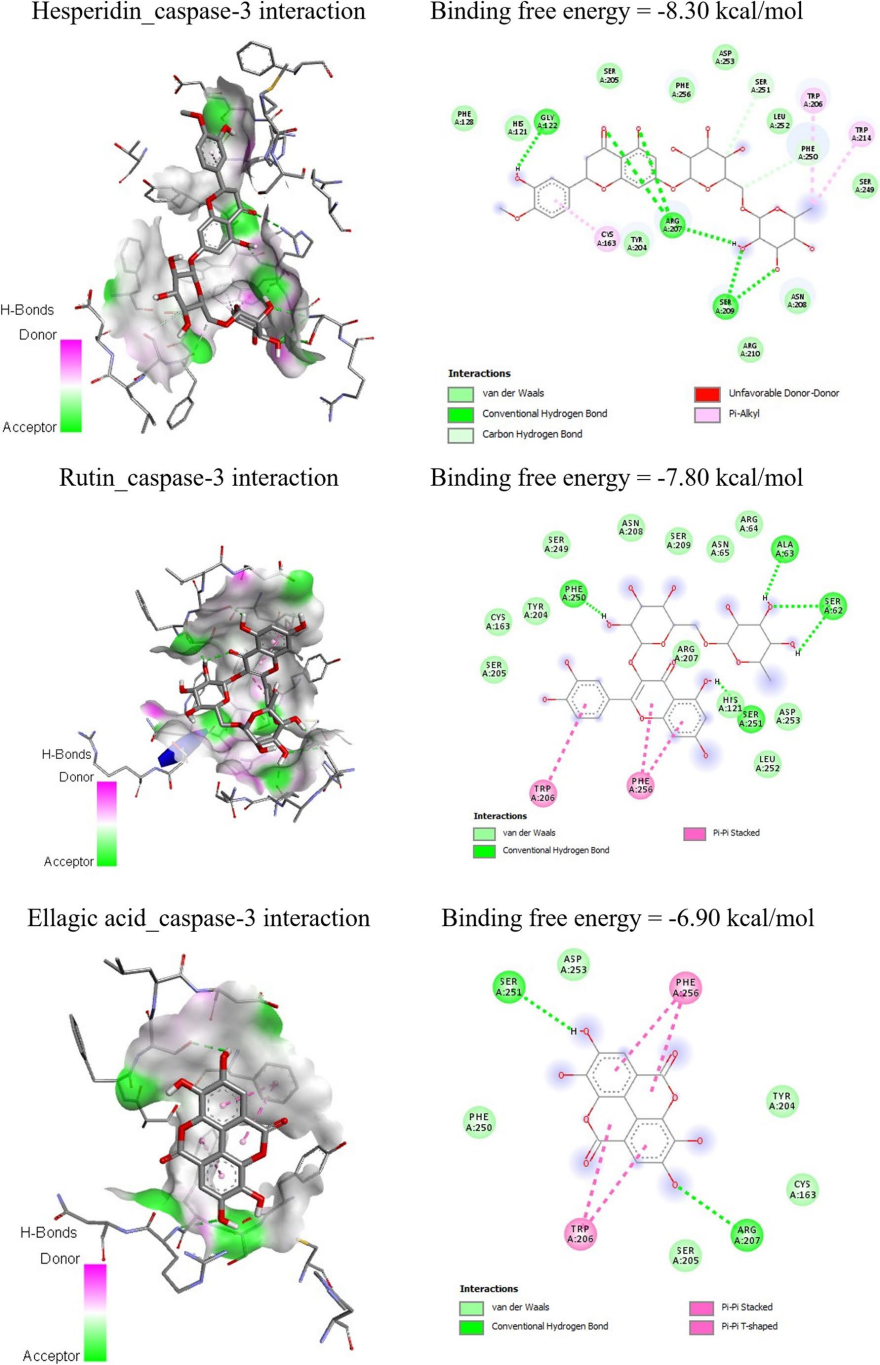
Molecular docking binding free energies of (**A**) hesperidin, (**B**) rutin, and (**C**) ellagic acid against caspase-3

Molecular docking served as an exploratory computational tool to evaluate possible interactions between the main MAE phenolics and target proteins (TNF-α and caspase-3). Table 5; Figs. 10 and 11 represent the molecular docking interactions of the ice plant’s bioactive ingredients with TNF-*α* and caspase-3. Hesperidin, rutin, and ellagic acid exhibited strong binding affinities to TNF‑α, with energies of − 7.05, − 6.90, and − 6.50 kcal/mol, respectively. Docking analysis revealed that hesperidin interacts with key TNF‑α residues, including CYS148, PRO178, PRO180, LYS181, ASP182, GLU188, and TYR193. In Table 6; Fig. 9B to 10B, the principal residues interacting with rutin are HIS99, LYS144, LYS218, ILE222, and THR223. Ellagic acid interacted with GLY145, GLN146, LYS218, and TYR219 residues in the TNF-*α* binding site with − 6.50 kcal/mol (Table 5; Fig. 9C to 10C).

**Table 5:** Molecular Docking binding free energies of ice plant’s bioactive ingredients against TNF-*α* and caspase-3

Ligands	Binding Free Energy (kcal/mol)	
	TNF-α	Caspase-3
1,2–15,16-Diepoxyhexadecane	−3.40	−4.20
1,2 Benzisothiazole, 3(hexahydro1Hazepin1yl),1,1dioxide	−5.40	−6.40
Caffeic acid	−5.10	−5.30
Catechin	−5.90	−6.90
Chlorogenic acid	−6.30	−6.80
Ellagic acid	−6.50	−6.90
Gallic acid	−4.70	−4.80
Hesperidin	−7.50	−8.30
Quercetin	−6.10	−6.60
Rutin	−6.90	−7.80

**Table 6:** Molecular Docking interactions of ice plant’s bioactive ingredients against TNF-*α*

Ligands	Residues	Hydrogen bonds	Charge	Hydrophobic interactions	Halogen
Hesperidin	CYS148	0	0	1	0
	PRO178	1	0	0	0
	PRO180	0	0	1	0
	LYS181	0	0	1	0
	ASP182	1	0	0	0
	GLU188	1	0	0	0
	TYR193	1	0	0	0
Rutin	HIS99	1	0	0	0
	LYS144	0	0	1	0
	LYS218	1	0	0	0
	ILE222	0	0	1	0
	THR223	1	0	0	0
Ellagic acid	GLY145	1	0	0	0
	GLN146	1	0	0	0
	LYS218	0	0	1	0
	TYR219	0	0	1	0

Regarding the molecular interaction with the caspase-3 binding site, hesperidin interacted with the TYR204, TRP206, SER209, and TRP214 residues, exhibiting a binding free energy of −8.30 kcal/mol (Table 7; Fig. 10A to 11A). As shown in Table 7; Fig. 10B to 11B, rutin interacts with the SER62, ALA63, TRP206, PHE250, SER251, and PHE256 residues, and the binding free energy is −7.80 kcal/mol, specifically with the caspase-3 binding site. Furthermore, ellagic acid demonstrated a binding affinity of −6.90 kcal/mol with caspase-3, interacting at residues LYS137, THR140, TYR195, and TYR197 (Table 7; Fig. 11C). Interactions observed with hesperidin, rutin and ellagic acid support their potential utility in therapeutic strategies.

**Table 7:** Molecular Docking interactions of ice plant’s bioactive ingredients against caspase-3

Ligands	Residues	Hydrogen bonds	Charge	Hydrophobic interactions	Halogen
Hesperidin	TYR204	0	0	1	0
	TRP206	0	0	1	0
	SER209	1	0	0	0
	TRP214	1	0	0	0
Rutin	SER62	1	0	0	0
	ALA63	1	0	0	0
	TRP206	0	0	1	0
	PHE250	1	0	0	0
	SER251	1	0	0	0
	PHE256	0	0	1	0
Ellagic acid	LYS137	0	0	1	0
	THR140	1	0	0	0
	TYR195	0	0	1	0
	TYR197	1	0	0	0

Docking results showed that hesperidin displayed the strongest binding energy with TNF-*α* and caspase-3 among the three biochemicals. Hesperidin’s binding energy is −7.05 kcal/mol and − 8.30 kcal/mol, respectively. Hesperidin exhibits stronger interactions with the caspase‑3 active site than with TNF‑α. Exchangeable Hydrogens on TNF-*α* show greater affinity for hesperidin while those on caspase-3 preferentially interact with rutin, and overall, more residues interact with hesperidin and rutin.

## Discussion

Qualitative HPLC results of the *M. crystallinum* extract confirmed the detection of eight key bioactive compounds including the prominent polyphenols quercetin, ellagic acid, rutin, hesperidin, caffeic acid, chlorogenic acid, catechin, and gallic acid. This composition provides a strong foundation for the observed effects, which align the known properties of flavonoids and phenolic acids as potent antioxidants. Their therapeutic efficacy is not attributed to a single compound, but rather to the synergistic biochemical interactions of their constituents, a concept supported by prior research on polyphenol combinations [25].

Previous reports by Rowida & Elsebaie [26] similarly identified multiple phenolics in *M. crystallinum*, with catechin being the most abundant, while Hamed et al. [27] demonstrated the presence of flavonoids, such as rutin and neo hesperidin in *M. forsskalei*, emphasizing the antioxidative properties of this genus. Although D-Pinitol- a widely recognized bioactive with antidiabetic, antioxidant, anti-inflammatory, anticancer, hepatoprotective, cardioprotective, and neuroprotective advantages were not quantified in the current work, its reported occurrence in *M. crystallinum* suggests it may synergize with other phytochemicals to enhance biological efficacy. Collectively, these phytoconstituents likely act as in combination to underpin the extract’s protective action.

CdCl₂ is a well-known environmental toxicant that significantly disrupts male reproductive function by impairing testicular structure, sperm quality, and hormonal balance [8]. In line with this, CdCl₂ administration in the current work resulted in a significant decrease in relative testis weight, indicative of testicular atrophy. Treatment with *M. crystallinum* extract effectively reversed this reduction particularly at higher doses (400 mg/kg), suggesting preservation of testicular tissue integrity, an effect comparable to that reported for polyphenol rich plants like *Vitis vinifera* [28]. This protective effect is associated to the antioxidant and anti-inflammatory actions of the plant extract [29].

CdCl₂ also significantly impaired sperm parameters including count, motility, viability, and velocity (VCL)- consistent with its ability to induce testicular oxidative stress, apoptosis, and impaired spermatogenesis [30]. Conversely, MAE treatment normalized these parameters in a dose dependent manner, with pretreatment showing superior efficacy. The improvement in linearity and wobble observed in MAE treated groups further reflect enhanced trajectory stability and energy efficiency, likely due to preserved mitochondrial function and membrane integrity [29]. These findings align with earlier studies demonstrating that antioxidant therapy restore sperm quality by suppressing reactive oxygen species (ROS) levels and preserving structural proteins [31–33].

CdCl₂ exposure also disrupted endocrine function by lowering LH and testosterone levels, reflecting hypothalamic-pituitary-gonadal axis suppression and Leydig cell dysfunction, resulting in reduced testosterone synthesis [34, 35]. Cadmium may additionally compete with zinc within sperm cells, resulting in impairing sperm morphology and viability [36]. Notably, molecular docking analysis in this study suggested direct interactions between CdCl₂ and the binding sites of FSHR, LHR, and androgen receptors, which may further explain the observed hormonal disruption. MAE treatment restored testosterone levels to values approximately14-fold higher than those observed in CdCl₂-only group, likely through multiple synergistic mechanisms. First, the contribution of saponins, which although undetected in the current HPLC profile, our recent study has confirmed their present in *M. crystallinum* extracts [37]. Saponins are known to elevate testosterone levels indirectly by stimulating LH release from the pituitary, which subsequently activate Leydig cells to secrete testosterone [38]. In parallel, the flavonoid constituents of MAE- particularly rutin, ellagic acid and quercetin may maintain androgen production by inhibiting an aromatase (17β-oestradiol aromatase), the key enzyme involved in testosterone to estrogen conversion [39]. This aromatase inhibiting effect aligns well with our findings and may contribute to the sustained elevation of circulating testosterone. The phytochemical profile of MAE also contributes to testicular recovery through several complementary mechanisms. The occurrence of gallic acid, rutin, ellagic acid and quercetin in MAE extract provides additional hormonal recovery. Gallic acid has shown to protect testicular tissue, reduce oxidative stress (OS), and enhance spermatogenesis in various toxicity models, including chemotherapy, phthalates, and nicotine induced damage [40]. Ellagic acid supplementation has similarly been reported to restore antioxidant defense system and correct hormonal balance in busulfan induced testicular damage [41]. Notably, the combination of ellagic acid and quercetin demonstrates enhanced bioactivity due to synergistic interactions beyond their individual effects [25]. Both compounds have also been shown to alleviate the hepatotoxicity through antioxidant, anti-inflammatory and metal-chelating mechanisms [42], which may extend to testicular detoxification in the context of Cd exposure. Importantly, our recently published study on *M. crystallinum* (ice plant) provides direct evidence of its detoxifying capabilities, showing a significant reduction in concentration of titanium dioxide nanoparticles in hepatic and renal tissues under OS conditions [37]. Also, in an unpublished work from our laboratory, MAE treatment markedly enhanced the clearance of CdCl_2_ accumulation in renal tissue compared to CdCl_2_ - treated group, lending further support to its metal-chelating and systemic detoxification properties. Collectively, these findings support the notion that the elevated testosterone level in MAE + CdCl_2_ - treated groups is not the result of a direct anabolic or stimulatory effect of the plant on steroidogenesis. Instead, they reflect the plant’s strong chemoprotective efficacy against acute Cd-induced testicular injury, thereby enabling testicular tissue to restore normal or even compensatory-testosterone production even under toxic conditions.

OS occurs when ROS production exceeds antioxidant defenses, leading to cellular damage. Excess ROS interacts with biomolecules, such as DNA, proteins and lipids, causing structural modifications and cell death. While ROS can arise from external factors like radiation, toxins and pollutants, they are also generated endogenously as byproducts of oxygen- dependent metabolic processes contributing to the pathogenesis of various human diseases [43]. OS- an established driver of male infertility- was confirmed by elevated MDA and suppressed antioxidant defenses (CAT, SOD, GPX and GSH) in Cd-treated set. These data align with Sheweita *et al*. [44], who investigated a similar antioxidant depletion in Cd-exposed rabbits. Antioxidant therapy has demonstrated a strong efficacy in mitigating OS-induced testicular dysfunction by neutralizing free radicals, preserving the integrity of the blood barrier of testis, and safeguarding sperm DNA from oxidative damage [27]. Likewise, MAE effectively restored antioxidant levels, exhibiting a protective effect comparable to that reported for individual antioxidants such as gallic acid and rutin. Earlier studies confirmed that gallic acid or selenium supplementation prior Cd exposure normalized testicular antioxidant activity like CAT, GPX and SOD and replenished GSH levels in rabbits [44]. Another study further confirmed rutin’s anti-apoptotic and antioxidant roles in reproductive protection [45]. In agreement with these observations, our HPLC findings (Table 1) verified the existence of both rutin and gallic acid in MAE, indicating that its antioxidant effect likely arises from the combined action of multiple phenolic compounds rather than a single compound. Thus, MAE’s antioxidant profile likely accounts for its ability to maintain sperm and testicular integrity.

Histopathological evaluation further confirmed that MAE preserved the architecture of the spermatogenic tubules, maintained germinal epithelium organization and prevented Cd-induced degeneration. Pretreatment offered the greatest protection, suggesting prophylactic advantage. These findings parallel protective roles reported for natural antioxidants such as, silymarin, resveratrol, and quercetin against Cd-induced testicular injury [46, 47].

At the molecular level, Cd exposure triggered apoptosis, as demonstrated by increased caspase-3 and TNF-*α* expression consistent with earlier reports on Cd- activated intrinsic apoptotic pathways [8, 48]. Moreover, Cd exposure is known to prompt inflammatory responses and tissue degeneration, often accompanied by increased pro- inflammatory cytokines, notably TNF-*α* [49]. The observed rise in TNF-*α* in Cd-treated rats in this study further confirms this inflammatory cascade, likely mediated by OS [50]. Mechanistically, OS stimulates NF-kB signaling [51], which enhances transcription of pro inflammatory cytokines, while concurrently promoting apoptosis by impairing mitochondrial function and disrupting the caspase cascade [54].

MAE treatment effectively countered these molecular disturbances by suppressing apoptosis and enhancing cellular survival pathways. Its antioxidant and anti-inflammatory potentials- particularly attenuation of IL-6, TNF-α, and IL-1β -have been previously documented [52, 53]. The molecular docking results provide a mechanistic insight into the *in vivo* antioxidant, anti-inflammatory and anti-apoptotic effects observed following treatment with MAE. Hesperidin, rutin, and ellagic acid displayed strong binding affinities toward TNF-α and caspase-3, particularly through hydrogen bonding and polar interactions with residue located in the active and regulatory regions, suggesting a potential inhibition of TNF-α signaling. Such interactions are consistent with the significant reduction in OS and inflammatory biomarkers reported *in vivo*, as TNF-α is a key mediator linking inflammation with oxidative damage.

Moreover, the pronounced affinity of hesperidin and rutin toward the caspase-3 binding site- especially involving residues associated with catalytic activity and substrate recognition-supports a plausible mechanism for apoptosis modulation. The superior binding energy of hesperidin to caspase-3 compared with TNF-α (−7.05 kcal/mol for TNF-α and − 8.30 kcal/mol for caspase-3) aligns with the observed *in vivo* attenuation of apoptotic markers and preservation of tissue architecture, indicating that stabilization of the caspase-3 active site may drive its protective efficiency. Complementary interactions were observed for rutin, implying possible synergistic inhibition [54–57]. Importantly, the ranking of docking scores (hesperidin ˃ rutin ˃ ellagic) mirrors the relative *in vivo* potency trends across antioxidant, anti-inflammatory, and anti-apoptotic parameters. This concordance strengthens the integration between computational predictions and experimental outcomes, reinforced the mechanistic relevance of docking analysis without necessitating additional computational experiments. Such approach is widely accepted as supportive evidence in phytochemical-based *in vivo* studies.

Together, these findings confirm that MAE exerts multilevel protection against Cd-induced reproductive damage by attenuating OS, suppressing inflammation, and inhibiting apoptosis-especially at 400 mg/kg dose.

## Limitations and Future Work

Despite the strong protective effects observed in this study, certain limitations should be acknowledged. 1- The sample: the relatively small sample size may reduce the statistical power and generalizability. Larger cohort would strengthen confidence in observed effects. 2. Exposure model: the study used an acute cadmium exposure mode which does not fully reflect chronic environmental or occupational conditions. 3. Functional fertility assessment: while sperm quality and hormonal profiles were evaluated, functional endpoints such as mating success and offspring viability were not evaluated. These are critical for confirming reproductive competence. 4. Bioactive compound specificity: while MAE demonstrated broad efficacy, the individual bioactive constituents responsible for protective effects were not isolated or tested separately. This makes it difficult to attribute mechanisms to distinct compounds. 5- safety and long-term effects: the study did not include long-term toxicity or safety profiling of the extract, which is essential before considering therapeutic applications. 6- mechanistic validation: while molecular docking provided preliminary mechanistic insights, *in vitro* studies and pathway specific assays are needed to confirm these interactions experimentally. Ultimately, clinical translation will be necessary to confirm MAE’ s therapeutic relevance in managing heavy metal-induced infertility.

## Conclusion

The data of this study strongly support the hypothesis that *M. crystallinum* (ice plant) may have a protective effect against CdCl₂-induced reproductive toxicity. MAE supplementation not only mitigates sperm damage, testicular atrophy, and hormonal imbalances but also enhances sperm motility, viability, and overall reproductive health. These beneficial effects are likely due to its antioxidant, endocrine-modulating, anti-apoptotic, and anti-inflammatory characteristics. Future studies should clarify the molecular mechanisms underlying MAE’s protective action, as well as its potential clinical applications in treating heavy metal-induced infertility.

## Supplementary Information

Below is the link to the electronic supplementary material.

Supplementary Material 1 (DOCX 283 KB)

## Data Availability

Upon request, the corresponding authors can provide the data.

## Abbreviations

ALH: Amplitude of lateral head displacement
ACEC: Animal Care and Ethics Committee
BCF: Beat cross frequency
BWG: Body weight gain
Cd: Cadmium
CdCl_2_: Cadmium chloride
CAT: Catalase
CASA: Computer-assisted Sperm System
FSHR: Follicle-stimulating hormone receptor
FSH: Follicle stimulating hormone
GPx: Glutathione peroxidase
H&E: Hematoxylin and eosin
HPLC: High-performance liquid chromatography
LH: Luteinizing hormone
LIN: Linearity
LHR: luteinizing hormone receptor
MAE: *M. crystallinum* aqueous extract
MDA: Malondialdehyde
MAD: Mean angular degree
*M. crystallinum*: *Mesembryanthemum crystallinum*
OS: Oxidative stress
ROS: Reactive oxygen species
GSH: Reduced glutathione
RTW: Relative testes weight
RT: Retention time
STR: Straightness
SOD: Superoxide dismutase
SDG: Sustainable development goals
TNF-α: Tumor necrosis factor-alpha
VAP: Velocity average path
VCL: Velocity Curved Line
VSL: Velocity straight line
WOB: Wobble
