# Correction: Protective Effects of *Mesembryanthemum Crystallinum* Extract Against Cadmium-Induced Reproductive Oxidative Stress: Experimental and Docking Evidence for a Sustainable Therapeutic Strategy

**DOI:** 10.1007/s12011-026-05058-w

**Published:** 2026-03-24

**Authors:** Hagar E. Mohammed, Ali El-Far, Shymaa Sobhy Mourad, Mai Alaa El-Dein, Menna H. E. Morsy, Mahmoud Bassiony, Shaimaa A. Hamouda

**Affiliations:** 1 Zoology Department, Faculty of Science, Arish University, North Sinai, Al-Arish, North Sinai 45511 Egypt; 2 Biochemistry Department, Faculty of Veterinary Medicine, Damnhour University, Damnhour, 22511 Egypt; 3 Zoology Department, Faculty of Science, Mansoura University, Mansoura, 35561 Egypt; 4 Faculty of Medicine, Alexandria University, Alexandria, Egypt

**Correction: Biological Trace Element Research**

10.1007/s12011-026-04975-0

The original version of this article contained a mistake. 

In this article the author’s name Mai Alaa El-Dein was incorrectly written as Mai Alaa.

The original article has been updated.

