# Supplementary material for: Protective Effects of Mesembryanthemum Crystallinum Extract Against Cadmium-Induced Reproductive Oxidative Stress: Experimental and Docking Evidence for a Sustainable Therapeutic Strategy
Source: Biol Trace Elem Res. 2026 Feb 4;204(6):4441–60. doi: 10.1007/s12011-026-04975-0 (PMC13157438; doi:10.1007/s12011-026-04975-0)
Supplement: Supplementary file 1 — Supplementary Material 1 (DOCX 283 KB) [file 12011_2026_4975_MOESM1_ESM.docx]

Supplemental 1. Experimental Group Design and Treatment Protocol.

| **Group** | **Treatment Type** | **Dose** | **Description** |
| --- | --- | --- | --- |
| 1 | Control | – | Normal, no cadmium or extract |
| 2 | CdCl₂ only | – | Toxicity group |
| 3 | MAE only | 200 mg/kg | Low-dose extract only |
| 4 | MAE only | 400 mg/kg | High-dose extract only |
| 5 | CdCl₂ + MAE (co-treatment) | 200 mg/kg | Low-dose extract given together with CdCl₂ |
| 6 | CdCl₂ + MAE (co-treatment) | 400 mg/kg | High-dose extract given together with CdCl₂ |
| 7 | MAE pre-treatment + CdCl₂ | 200 mg/kg | Low-dose extract given for 1 week before Cd exposure |
| 8 | MAE pre-treatment + CdCl₂ | 400 mg/kg | High-dose extract given for 1 week before Cd exposure |
